# Supplementary material for: The response to selection in Glycoside Hydrolase Family 13 structures: A comparative quantitative genetics approach
Source: PLoS One. 2018 Apr 26;13(4):e0196135. doi: 10.1371/journal.pone.0196135 (PMC5919626; doi:10.1371/journal.pone.0196135)
Supplement: S1 File — Supplementary methods (A) and results (B) testing the feasibility of the of the linear mixed model and Bayesian mixed model implementations. (PDF) [file pone.0196135.s001.pdf]

Supplementary material for "The response to selection in Glycoside Hydrolase Family 13 structures: A comparative quantitative genetics approach"

Jose Sergio Hleap[1,] and Christian Blouin[2,3]

Keywords: Geometric analysis, homolog variation, relative deformation, protein structure engineering, protein structure evolution

[2]Department of Human Genetics, McGill University

[3]Department of Computer Science, Dalhousie University

*Corresponding author:

Department of Human Genetics

McGill University

740 Dr Penfield Ave

Office 7209

Montreal, QC H3A 0G1

A Supplementary Material and Methods

A.1 Shape Simulation

The phenotype was simulated following equation 5 in the main paper, where the phylogenetic effects (a) were simulated using the `rbv` function in the `MCMCglmm` `R` package [13] to create a matrix of randomly generated multivariate normal phylogenetic effects. The generation of phylogenetic effects was constrained with a known $p \times p$ genetic matrix (G) that was drawn from an inverse Wishart distribution with a scale matrix with the digit 1 in the diagonal and 0.5 in the off diagonal entries. The input tree for the simulation of the phylogenetic effects was generated by the functions `compute.brtime` and `rtree` of the `ape` `R` package [36]. The error component (e) was built by performing a Cholesky factorization of a known $p \times p$ covariance matrix (E) and multiplying the decomposed matrix with a matrix of random values of shape $n \times p$. The known E matrix was also drawn from an inverse Wishart distribution, and its scale matrix contained the digit 1 in the diagonal and 0.1 in the off diagonal. Summarizing, the simulation was performed by:

1. Creating the known phylogenetic (G) and error (E) covariance matrices
2. Using G to constrain the simulation of the phylogenetic effects (a), simulating the $n \times p$ matrix a given a random tree
3. Incorporating the desired covariation E into a $n \times p$ random matrix
4. Adding the error and phylogenetic effects

Here, the number of observations (n) and the number of variables/traits (p) were controlled.

A.1.1 Including a within group component in the simulation

To include a within group component to the simulation the same approach used before was applied, but a dynamic term was included in the simulation and the model. This term was performed as follows:

1. Generating a multivariate normal $p \times r$ matrix O, where r is the number of repetitions.

2. Correlating the variables with a known covariance matrix M (drawn from an inverse Wishart distribution), by means of Cholesky decomposition.

3. Populating an MD matrix $(n * r) \times p$, with the correlated matrix by repeating it n times.

4. Generating a multivariate normal $(n * r) \times p$ matrix of independent effects.

5. Adding the MD matrix with the independent effects.

This within group effect can be abstracted as a dynamic component in the analysis of MD simulations throughout homologs.

A.2 GH13 dataset

We gather 386 protein structures classified as GH13. Given the computational costs of molecular dynamic (MD) simulations, a subset of 35 structures were selected.

One of the 35 structures failed during the MD simulation. A final set of 34 protein structures (Table 1) was used in further analyses.

A.3 Testing Linear Mixed Models implementations

The estimations of G and E matrices with the Lynch's PMM model were performed using the Lynch's algorithm implementation in the `R` package `ape` [36]. The Bayesian Generalized Linear Mixed Model (BGLMM) estimations of G and E were performed using the `R` package for BGLMM `MCMCglmm` [13].

A.4 Testing the accuracy of the estimations

To test the extent of the bias in the estmations of G and E, the mean correlation and corresponding p-values of the Cheverud's Random Skewer (RS) test [6, 7] implemented in the `R` package `phytools` [41] were used. Works such as Bégin et al. [3] have contentions about any given covariance matrix comparison methods; however, Cheverud's test is better suited for the framework under study. It introduces random vectors of change and compares the correlation of resulting vectors. This is in line with the quantitative genetic framework as in equation 3 in the main paper. It is also better suited for comparative studies than other tests of equality such as Anderson's maximum likelihood test of equality of covariances [2] or the common principal component (CPC) analysis [38]. Those tests have big biases given the sample size and the number of traits. Steppan [47] showed a positive relationship between the number of traits and the likelihood of rejecting equality.

Table 1: Subset of the α-Amylase dataset. PDB codes, taxonomic information, Enzyme commission code and reference of the 34 structures used to estimate the pooled-within covariance matrix

**PDB code**	**Species**	**EC code**	**Mutation**	**Reference**
1CGY	*Bacillus circulans*	2.4.1.19	Y195W	[37]
1E3X	*Bacillus amyloliquefaciens*	3.2.1.1	Chimeric	[4]
1G5A	*Neisseria polysaccharea*	2.4.1.4	None	[46]
1GVI	*Thermus* sp.	3.2.1.54*	None	[24]
1J0H	*Geobacillus stearothermophilus* TRS40	3.2.1.135	None	[16]
1KB3	*Homo sapiens*	3.2.1.1	R195A	[35]
1KXH	*Pseudoalteromonas haloplanctis*	3.2.1.1	D174N†	[1]
1M53	*Klebsiella* sp. LX3	5.4.99.11	None	[52]
1SMA	*Thermus* sp. IM6501	3.2.1.133	None	[22]
1TMQ	*Tenebrio molitor*	3.2.1.1	None	[48]
1UA7	*Bacillus subtilis*	3.2.1	N356Q	[21]
1UD3	*Bacillus* sp. KSM-K38	3.2.1.1	N289H	[34]
1VJS	*Bacillus licheniformis*	3.2.1.1	None‡	[19]
1W9X	*Bacillus halmapalus*	3.2.1.1	None	[8]
1WZL	*Thermoactinomyces vulgaris* R-47	3.2.1.135	R469L	[31]
1ZJA	*Pseudomonas mesoacidophila*	5.4.99.11	None	[39]
2DIE	*Bacillus* sp. KSM-1378	3.2.1.1	None	[43]
2FH8	*Enterobacter aerogenes*	3.2.1.41	G680L/V882L	[30]
2TAA	*Aspergillus oryzae*	3.2.1.1	None	[28]
2WAN	*Bacillus acidopullulyticus*	3.2.1.41	None⨿	[49]
2Y4S	*Hordeum vulgare*	3.2.1.41	None	[50]
2Z1K	*Thermus thermophilus HB8*	3.2.1.41	NA	NA
2ZE0	*Geobacillus* sp. HTA-462	3.2.1.20	None	[44]
2ZIC	*Streptococcus mutans*	3.2.1.70	N536L	[17]
3AXH	*Saccharomyces cerevisiae*	2.1.1.64/3.2.1.10	E277A	[51]
3CZK	*Xanthomonas axonopodis* pv. glycines	3.2.1.48	E322Q	[23]
3DC0	*Bacillus* sp. KR-8104	3.2.1.1	NA	NA
3EDE	*Flavobacterium* sp. 92	3.2.1.54	T49P	[5]
3GBD	*Serratia plymuthica*	5.4.99.11	None	[40]
3UEQ	*Neisseria polysaccharea*	2.4.1.4	None	[11]
3VM5	*Oryzias latipes*	3.2.1.1*	None	[32]
3VM7	*Malbranchea cinnamomea*	3.2.1.73*	None	[14]
4E2O	*Geobacillus thermoleovorans* CCB_US3_UF5	3.2.1.1	None⊎	[33]
4GI6	*Rhizobium* sp. MX-45	5.4.99.11	F164L	[25]

* EC number derived from enzyme name using BRENDA [42]

† Inactive mutant

‡ Thermostable α-amylase

⨿ A588 CYS Modelled as oxidised CYS (CSX)

⊎ Truncated

NA Not provided by the RCSB PDB or secondary sources

B Supplementary results

B.1 Computational infeasibility of the full comparative approach

In a multivariate scenario, both REML and Bayesian methods have a computational chokepoint on the number matrix operations needed to estimate a large number of parameters. As the matrix grows bigger, in both number of individuals (n) and number of traits (p), the computation becomes untractable in terms of memory and time. For p traits, $p(p+1)/2$ covariance components need to be estimated per random effect, and thus the estimation burden increases quadratically [29, 18]. Table 2 shows the time and memory spent in a simulation fixing n to 100 and varying p. The estimation was performed using Lynch's PMM model as implemented in the `R` package `ape` [36], where many matrix inversions and Kronecker products are involved [see 27, for details]. This computation was performed in a PC Intel®Xeon®CPU E5-2620 v2 @ 2.10GHz Intel i3 3.10GHz 128 Gb 2x hexa core processor.

Table 2 shows that at 16 traits over 300 Mb of memory and over 9.4 hours are required to compute the model. The behaviour with 32 traits is erratic. In the current simulation the computation cannot be performed since the memory requirements are too high. The problem scales up quickly, not only with the number of traits, but also with the number of individuals. This trend is due to computation of the inverse of the relationship and the identity matrices. The computation of these matrices take the most time (≈94%) and memory (≈60%). Also, the time and memory required to compute the Kronecker product of these very large matrices scale up quickly with the

Table 2: Feasibility of the phylogenetic mixed model (PMM). Memory, time and accuracy of the PMM using Lynch's and Bayesian approaches. RS_A correspond to the random skewer test for the phylogentic covariance and RS_E for the residual. Bold values indicate correlation of random skewers greater than 0.9 and significant.

**Method**	**Traits**	**Time (secs)**	**Memory (Mb)**	RS_A		RS_E	
				p-val	ρ	p-val	ρ
Lynch	2	113.44	13.9	0.109	0.955	0.036	**0.997**
	4	1132.34	27	0.004	**0.952**	0.000	**0.995**
	8	1780.78	77.7	0.003	0.878	0.000	**0.997**
	16	34159.34	276.8	0.000	0.888	0.000	**0.998**
BGLMM	2	35.94	50.5	0.103	0.948	0.024	**0.997**
	4	110.36	62.2	0.002	**0.971**	0.001	**0.997**
	8	624.88	107.8	0.000	**0.940**	0.000	**0.994**
	16	2508.96	310	0.000	0.897	0.000	**0.995**
	32	9777.48	1110	0.000	**0.904**	0.000	**0.995**

dimensions of the input matrix. It can take up almost a third of the spent memory, showing the dependency between complexity and both n and p. The reliability of the estimates is also affected. The sample size needs to be increased in order to estimate the covariance matrices with confidence. However, by increasing the sample size the computation becomes more complex. To test the extent of the bias Table 2 shows the mean correlation and corresponding p-values of the Cheverud's Random Skewer (RS) test [6, 7].

Surprisingly, despite the expected instability of the matrix estimation given the sample size, most of the estimations were highly correlated (>0.70) and significant. It is important to state that the (RS) test does not evaluate equality of the covariances (in fact most covariances were very dissimilar). However, the overall response

to a vector of selection is highly correlated. For the purposes of this work, the matrix equality in response to disturbances is more relevant than the exact match between the covariance matrices' values. This is because the response to disturbances expressed in the RS test follows the same framework as the expression detailed in equation 3 in the main paper.

B.1.1 Bayesian solution to the memory requirements

Given that Bayesian generalized linear mixed models (BGLMM) use Markov chain Monte Carlo (MCMC) simulations and usually Gibbs sampling, the memory requirements should lower significantly. However, Table 2 show the same trend on a different scale when using the `R` package for BGLMM `MCMCglmm` [13].

We simulated up to 32 traits using approximately 2 hours in the bigger dataset when the sample size and the number of MCMC iterations were held constant. The results show that it was not the memory but the time that benefited from the Bayesian approach since over 1Gb was used (Table 2). However, this memory requirement can be lowered if fewer MCMC iterations are performed. Nevertheless, lowering the number of iterations can only be done on a per-case basis since the convergence has to be guaranteed.

Data in Table 2 describe the accuracy of this approach. With these particular data, there is higher or similar accuracy to that of Lynch's approach while being faster when the number of traits is high. With the given dataset, when more than 32 variables were analysed, `MCMCglmm` [13] reported ill-conditioned priors when completely flat priors (identity matrices) were used. With this in mind, and the fact

that on 32 traits the memory requirements remain over 1Gb, a new approach was required.

B.1.2 Sample size effect in the estimation

It is known that the sample size is also an issue in the estimations of G especially when the number of traits increases. To test this assumption, we simulated and measured time and memory usage for the estimations following the same strategy used as before. In this case, the simulation had a fixed p equal to 8 and varying levels of n: low (16 observations), medium (64 observations), and high (256 observations). Table 3 shows the requirements in time and memory, as well as the average accuracy and the standard deviation for 10 replicates. The estimated mean is slightly biased resulting in a decrease in the mean.

Table 3: Effect of sample size in the phylogenetic mixed model(PMM). Average (± standard deviation) memory, time and accuracy of the PMM using Lynch's and Bayesian approaches. RS_A corresponds to the random skewer test for the phylogenetic covariance and RS_E for the residual.

**Method**	**Samples**	**Time (secs.)**	**Memory(Mb)**	RS_A	RS_E
	16	43.76±53.77	15.77±0.05	0.72±0.26	0.85±0.26
Lynch	64	2943.23±2746.06	37±0	0.72±0.24	0.98±0.04
	256	111986.44±53576.95	444.63±0.05	0.71±0.26	1±0
	16	70.41±7.8	100.62±1.96	0.67±0.2	0.86±0.25
BGLMM	64	343.31±65.44	102.82±9.73	0.7±0.12	0.9±0.31
	256	1198.12±100.73	123.06±37.9	0.78±0.15	1±0

It is expected that accuracy increases with sample size. However, Table 3 shows that this is only significantly true for the residual matrices. This phenomenon might

be attributable to the differences in the scaling structure in the original simulated matrices.

Data from Table 3 also suggest that the cost in time and memory can be prohibitive. However, a greater problem is that obtaining sample sizes of over 200 protein structures is not always possible. One possibility to increase the sample size is to include snapshot structures from molecular dynamic simulations of each of the homologs. This approach also introduces an extra component to the variance: the within group (within homolog/species) component. Given that this component is actually of interest, it is worthy of being assessed separately.

B.2 Beyond the OTUs: partitioning the variance within taxonomic units

We know by the decomposition of the phenotype into its components that the phenotypic variance can be explained by the genetic, environmental, and interaction components. It is also known that if repeated measures of a trait are available, the variance can be further partitioned into a third component. In general, comparative evolutionary biology, such components, may include differences among populations, phenotypic plasticity, sampling variation, instrument-related error, physiological state, variation related to age, sex, season, or time of day, among others [20]. All these sources of variations greatly depend on the way the sampling was done and the trait in hand. By including a within-group, within individual in traditional quantitative genetics, or within species in comparative studies, nuisance parameters can be dealt with. Many studies have shown the importance of dealing with measurement

errors and deviations from the between-group analyses [15, 20, 9, 12, 10, 45]. Therefore the within-group analysis is ideal. In protein structures, repeated measures can be taken as snapshots of molecular dynamic simulations of a protein in solution (refer to methods for details), thereby adding a partition to the variance. In this set up, another variable is added to the model:

$$\bar{z} = Xb + Za + e + m \tag{1}$$

where m is the matrix of individual effects or effects of the dynamics of a protein.

This approach has an application in structural biology since it allows partitioning the structural variation into:

1. Phylogenetic component: This component will provide information about the evolutionary constraint in the protein structure. Therefore, it can be used to inform decisions of protein engineering, structural constraints in bioinformatics, etc...

2. Dynamic component: This component provides information about the thermodynamic constraints in a set of proteins. It can be used as before to guide within species protein engineering and the analysis of the dynamics of a given fold.

3. Residual component: This will encompass all other components of the phenotype including noise, different sources of error inclusive of measurement error, and other environmental factors.

Table 4: Accuracy and feasibility of the two random effect PMM. Memory (Mb), time (sec) and accuracy (random skewer correlation) of the PMM using a Bayesian approach with two random effects. RS_A corresponds to the random skewer test for the phylogenetic covariance, RS_M for the dynamic component, and RS_E for the residual.

**Method**	**Traits**	**Time (hours)**	**Memory (Mb)**	RS_A		RS_M		RS_E	
				p-val	ρ	p-val	ρ	p-val	ρ
BGLMM	2	1.497	185.1	0.033	0.992	0.077	0.963	0.227	0.758
	4	3.635	255.1	0.005	0.968	0.003	0.958	0.095	0.681
	8	11.1946	457.4	0.000	0.947	0.001	0.868	0.060	0.554
	16	40.562	1184.4	0.000	0.931	0.000	0.835	0.029	0.450

This approach is ideal since it allows one to dissociate the phylogenetic (evolutionary) variability from dynamic variability. Liu and Bahar [26] show a correlation between sequence evolution and dynamics. However, in their approach both entities can be confounded by permanent effects created by descent. Ideally, this bias should be stripped to avoid an artificially increased estimation of the correlation. Using the linear mixed model to estimate these components implies the inclusion of extra parameters. To test the computational complexity and the accuracy, a simulation including this component was performed (refer to Methods for details). The results can be seen in Table 4.

With the inclusion of extra parameters there is a significant increase in the time and memory required for the estimation. However, the high accuracy in the phylogenetic covariance matrix estimation is surprising since a more unstable estimation was expected. The accuracy was anticipated to diminish quickly with the number of variance-covariance components to be estimated. For 3 random effects, there are

$\frac{3q(q+1)}{2}$ variance-covariance components that have to be estimated. This complexity makes the estimation of parameters unstable and intractable. However, data in Table 4 show high correlation values for the phylogenetic and acceptable values for the dynamic component. In the case of the residual value, most correlations were low and non significant. This is also an interesting observation since the trend seen in previous sections showed that an accurate estimation of the residual matrix was more feasible than the phylogenetic one.

Despite an improvement in accuracy, it can be seen that the time and memory required for the computation are still prohibitive; therefore, when q and n are high, another approximation is needed.

B.3 Fitness landscape in the GH13 dataset

To infer the source and target structures, a fitness landscape must be defined. In this paper we computed the average $\Delta G^{\circ}_{unfold}$ per residue as $\Delta \hat{G^{\circ}_{unfold}} = \frac{\Delta G^{\circ}_{unfold}}{n}$, n being the number of residues. With this $\Delta \hat{G^{\circ}_{unfold}}$ as proxy for fitness we can try to explore the fitness surface. To do this, we used the first two principal components of a PCA analysis of the shapes as X and Y axes; $\Delta \hat{G^{\circ}_{unfold}}$ in the Z axis.

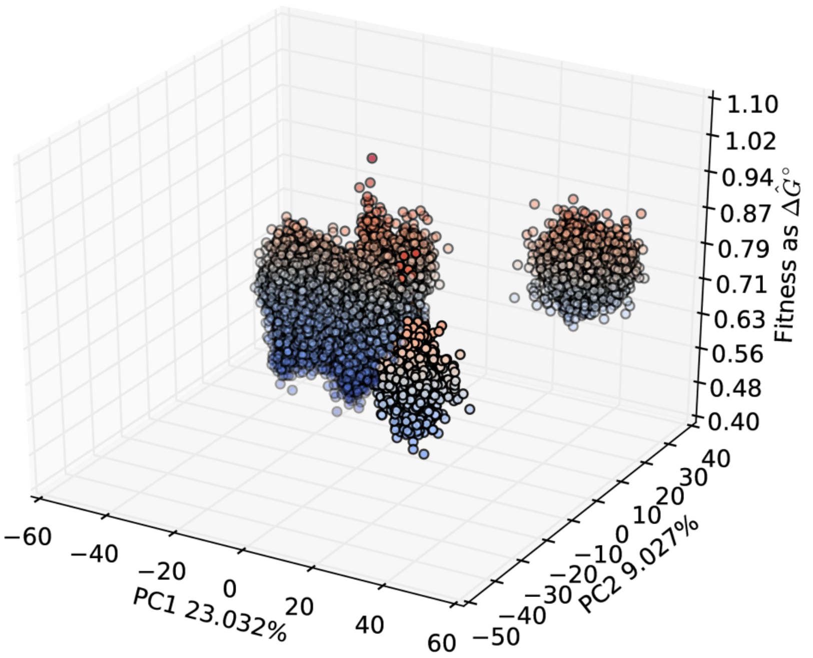

Figure 1: Fitness surface of the MD simulations in the 34 structures dataset. Fitness in the Z axis is defined as $\Delta\hat{G}^{\circ}$

References

[1] Nushin Aghajari, Michel Roth, and Richard Haser. Crystallographic evidence of a transglycosylation reaction: ternary complexes of a psychrophilic α-amylase. *Biochemistry*, 41(13):4273–4280, 2002.

[2] Theodore Wilbur Anderson. *An introduction to multivariate statistical analysis*, volume 2. Wiley New York, 1958.

[3] M. Bégin, D. A. Roff, and V. Debat. The effect of temperature and wing morphology on quantitative genetic variation in the cricket gryllus firmus, with an appendix examining the statistical properties of the jackknife–manova method of matrix comparison. *Journal of evolutionary biology*, 17(6):1255–1267, 2004. doi: 10.1111/j.1420-9101.2004.00772.x.

[4] Andrzej M. Brzozowski, David M. Lawson, Johan P. Turkenburg, Henrik Bisgaard-Frantzen, Allan Svendsen, Torben V. Borchert, Zbigniew Dauter, Keith S. Wilson, and Gideon J. Davies. Structural analysis of a chimeric bacterial α-amylase. high-resolution analysis of native and ligand complexes. *Biochemistry*, 39(31):9099–9107, 2000. doi: 10.1021/bi0000317.

[5] Stefan Buedenbender and Georg E Schulz. Structural base for enzymatic cyclodextrin hydrolysis. *J. Mol. Biol.*, 385(2):606–17, 2009. doi: 10.1016/j.jmb.2008.10.085.

[6] J. M. Cheverud. Quantitative genetic analysis of cranial morphology in the cotton-top (saguinus oedipus) and saddle-back (s. fuscicollis) tamarins. *Journal of Evolutionary Biology*, 9(1):5–42, 1996.

[7] James M. Cheverud and Gabriel Marroig. Comparing covariance matrices: random skewers method compared to the common principal components model. *Genetics and Molecular Biology*, 30(2):461–469, 2007.

[8] Gideon J. Davies, A. Marek Brzozowski, Zbigniew Dauter, Michael D. Rasmussen, Torben V. Borchert, and Keith S. Wilson. Structure of a bacillus

halmapalus family 13 α-amylase, bha, in complex with an acarbose-derived nonasaccharide at 2.1ă resolution. *Acta Crystallographica Section D*, 61(2):190–193, Feb 2005. doi: 10.1107/S0907444904027118. URL http://dx.doi.org/10.1107/S0907444904027118.

[9] Joseph Felsenstein. Comparative methods with sampling error and within-species variation: contrasts revisited and revised. *The American Naturalist*, 171(6):713–725, 2008.

[10] László Z Garamszegi and Anders P. Møller. Effects of sample size and intraspecific variation in phylogenetic comparative studies: a meta-analytic review. *Biological Reviews*, 85(4):797–805, 2010.

[11] Frédéric Guérin, Sophie Barbe, Sandra Pizzut-Serin, Gabrielle Potocki-Véronèse, David Guieysse, Valérie Guillet, Pierre Monsan, Lionel Mourey, Magali Remaud-Siméon, Isabelle André, and Samuel Tranier. Structural investigation of the thermostability and product specificity of amylosucrase from the bacterium deinococcus geothermalis. *J. Biol. Chem.*, 287(9):6642–54, 2012. doi: 10.1074/jbc.M111.322917.

[12] J. D. Hadfield and S. Nakagawa. General quantitative genetic methods for comparative biology: phylogenies, taxonomies and multi-trait models for continuous and categorical characters. *Journal of evolutionary biology*, 23(3):494–508, 2010.

[13] Jarrod D. Hadfield. Mcmc methods for multi-response generalized linear mixed models: the mcmcglmm r package. *Journal of Statistical Software*, 33(2):1–22, 2010.

[14] Peng Han, Peng Zhou, Songqing Hu, Shaoqing Yang, Qiaojuan Yan, and Zhengqiang Jiang. A novel multifunctional α-amylase from the thermophilic fungus *Malbranchea cinnamomea*: Biochemical characterization and three-dimensional structure. *Applied Biochemistry and Biotechnology*, 2(170):420–435, 2013. doi: 10.1007/s12010-013-0198-y.

[15] Luke J. Harmon and Jonathan B. Losos. The effect of intraspecific sample size on type i and type ii error rates in comparative studies. *Evolution*, 59(12): 2705–2710, 2005.

[16] Hironori Hondoh, Takashi Kuriki, and Yoshiki Matsuura. Three-dimensional structure and substrate binding of bacillus stearothermophilus neopullulanase. *Journal of molecular biology*, 326(1):177–188, 2003.

[17] Hironori Hondoh, Wataru Saburi, Haruhide Mori, Masayuki Okuyama, Toshitaka Nakada, Yoshiki Matsuura, and Atsuo Kimura. Substrate recognition mechanism of α-1, 6-glucosidic linkage hydrolyzing enzyme, dextran glucosidase from *Streptococcus mutans*. *Journal of molecular biology*, 378(4):913–922, 2008. doi: 10.1016/j.jmb.2008.03.016.

[18] David Houle. Numbering the hairs on our heads: the shared challenge and promise of phenomics. *Proc. Natl. Acad. Sci. U.S.A.*, 107 Suppl 1:1793–9, 2010. doi: 10.1073/pnas.0906195106.

[19] Kwang Yeon Hwang, Hyun Kyu Song, Changsoo Chang, Jungkyu Lee, S. Y. Lee, K. K. Kim, Senyon Choe, Robert M. Sweet, and S. W. Suh. Crystal

structure of thermostable alpha-amylase from bacillus licheniformis refined at 1.7 a resolution. *Molecules and cells*, 7(2):251–258, 1997.

[20] Anthony R Ives, Peter E Midford, and Theodore Garland. Within-species variation and measurement error in phylogenetic comparative methods. *Syst. Biol.*, 56(2):252–70, 2007. doi: 10.1080/10635150701313830. URL http://sysbio.oxfordjournals.org/content/56/2/252.abstract.

[21] Masayuki Kagawa, Zui Fujimoto, Mitsuru Momma, Kenji Takase, and Hiroshi Mizuno. Crystal structure of *Bacillus subtilis* α-amylase in complex with acarbose. *Journal of Bacteriology*, 185(23):6981–6984, 2003. doi: 10.1128/JB.185.23.6981-6984.2003.

[22] Jeong-Sun Kim, Sun-Shin Cha, Hyun-Ju Kim, Tae-Jip Kim, Nam-Chul Ha, Sang-Taek Oh, Hyun-Soo Cho, Moon-Ju Cho, Myo-Jeong Kim, Hee-Seob Lee, et al. Crystal structure of a maltogenic amylase provides insights into a catalytic versatility. *Journal of Biological Chemistry*, 274(37):26279–26286, 1999.

[23] Myung-Il Kim, Hong-Suk Kim, Jin Jung, and Sangkee Rhee. Crystal structures and mutagenesis of sucrose hydrolase from xanthomonas axonopodis pv. glycines: insight into the exclusively hydrolytic amylosucrase fold. *J. Mol. Biol.*, 380(4):636–47, 2008. doi: 10.1016/j.jmb.2008.05.046.

[24] Hee-Seob Lee, Min-Sung Kim, Hyun-Soo Cho, Jung-In Kim, Tae-Jip Kim, Ji-Hye Choi, Cheonseok Park, Heung-Soo Lee, Byung-Ha Oh, and Kwan-Hwa Park. Cyclomaltodextrinase, neopullulanase, and maltogenic amylase are nearly

indistinguishable from each other. *Journal of Biological Chemistry*, 277(24): 21891–21897, 2002. doi: 10.1074/jbc.M201623200.

[25] Alexandra Lipski, Hildegard Watzlawick, Stephanie Ravaud, Xavier Robert, Moez Rhimi, Richard Haser, Ralf Mattes, and Nushin Aghajari. Mutations inducing an active-site aperture in rhizobium sp. sucrose isomerase confer hydrolytic activity. *Acta Crystallographica Section D: Biological Crystallography*, 69(2):298–307, 2013. doi: 10.1107/S0907444912045532.

[26] Ying Liu and Ivet Bahar. Sequence evolution correlates with structural dynamics. *Mol. Biol. Evol.*, 29(9):2253–63, 2012. doi: 10.1093/molbev/mss097.

[27] Michael Lynch. Methods for the analysis of comparative data in evolutionary biology. *Evolution*, pages 1065–1080, 1991.

[28] Yoshiki Matsuura, Masami Kusunoki, Wakako Harada, and Masao Kakudo. Structure and possible catalytic residues of taka-amylase a. *Journal of Biochemistry*, 95(3):697–702, 1984.

[29] Karin Meyer and Mark Kirkpatrick. Perils of parsimony: properties of reduced-rank estimates of genetic covariance matrices. *Genetics*, 180(2):1153–66, 2008. doi: 10.1534/genetics.108.090159.

[30] Bunzo Mikami, Hiroyuki Iwamoto, Dominggus Malle, Hye-Jin Yoon, Elif Demirkan-Sarikaya, Yoshihiro Mezaki, and Yoshio Katsuya. Crystal structure of pullulanase: evidence for parallel binding of oligosaccharides in the active site.

Journal of molecular biology, 359(3):690–707, 2006. doi: 10.1016/j.jmb.2006.03.058.

[31] Masahiro Mizuno, K.azuhiro Ichikawa, Takashi Tonozuka, Akashi Ohtaki, Yoichiro Shimura, Shigehiro Kamitori, Atsushi. Nishikawa, and Yoshiyuki Sakano. Mutagenesis and structural analysis of thermoactinomyces vulgarius r-47 α-amylase ii (tvaii). *Journal of Applied Glycoscience*, 52(3):225–231, 2005. doi: 10.5458/jag.52.225.

[32] Kimihiko Mizutani, Mayuko Toyoda, Yuichiro Otake, Soshi Yoshioka, Nobuyuki Takahashi, and Bunzo Mikami. Structural and functional characterization of recombinant medaka fish alpha-amylase expressed in yeast pichia pastoris. *Biochimica et Biophysica Acta (BBA)-Proteins and Proteomics*, 1824(8):954–962, 2012. doi: 10.1016/j.bbapap.2012.05.005.

[33] Sook-Chen Mok, Aik-Hong Teh, Jennifer A. Saito, Nazalan Najimudin, and Maqsudul Alam. Crystal structure of a compact α-amylase from *Geobacillus thermoleovorans*. *Enzyme and microbial technology*, 53(1):46–54, 2013. doi: 10.1016/j.enzmictec.2013.03.009.

[34] Tsuyoshi Nonaka, Masahiro Fujihashi, Akiko Kita, Hiroshi Hagihara, Katsuya Ozaki, Susumu Ito, and Kunio Miki. Crystal structure of calcium-free α-amylase from bacillus sp. strain ksm-k38 (amyk38) and its sodium ion binding sites. *Journal of Biological Chemistry*, 278(27):24818–24824, 2003.

[35] Shin Numao, Robert Maurus, Gary Sidhu, Yili Wang, Christopher M. Overall, Gary D. Brayer, and Stephen G. Withers. Probing the role of the chloride ion in

the mechanism of human pancreatic α-amylase. *Biochemistry*, 41(1):215–225, 2002.

[36] Emmanuel Paradis, Julien Claude, and Korbinian Strimmer. Ape: Analyses of phylogenetics and evolution in r language. *Bioinformatics*, 20:289–290, 2004. doi: 10.1093/bioinformatics/btg412.

[37] Dirk Penninga, Boris Strokopytov, Henrieette J. Rozeboom, Catherine L. Lawson, Bauke W. Dijkstra, Jack Bergsma, and Lubbert Dijkhuizen. Site-directed mutations in tyrosine 195 of cyclodextrin glycosyltransferase from bacillus circulans strain 251 affect activity and product specificity. *Biochemistry*, 34(10): 3368–3376, 1995.

[38] Patrick C. Phillips and Stevan J. Arnold. Hierarchical comparison of genetic variance-covariance matrices. i. using the flury hierarchy. *Evolution*, 53(5):1506–1515, 1999. ISSN 00143820. URL http://www.jstor.org/stable/2640896.

[39] Stéphanie Ravaud, Xavier Robert, Hildegard Watzlawick, Richard Haser, Ralf Mattes, and Nushin Aghajari. Trehalulose synthase native and carbohydrate complexed structures provide insights into sucrose isomerization. *J. Biol. Chem.*, 282(38):28126–36, 2007. doi: 10.1074/jbc.M704515200.

[40] Stéphanie Ravaud, Xavier Robert, Hildegard Watzlawick, Richard Haser, Ralf Mattes, and Nushin Aghajari. Structural determinants of product specificity of sucrose isomerases. *FEBS Lett.*, 583(12):1964–8, 2009. doi: 10.1016/j.febslet.2009.05.002.

[41] Liam J. Revell. phytools: an r package for phylogenetic comparative biology (and other things). *Methods in Ecology and Evolution*, 3(2):217–223, 2012.

[42] Ida Schomburg, Antje Chang, Christian Ebeling, Marion Gremse, Christian Heldt, Gregor Huhn, and Dietmar Schomburg. Brenda, the enzyme database: updates and major new developments. *Nucleic acids research*, 32(suppl 1): D431–D433, 2004. doi: 10.1093/nar/gkh081.

[43] Tsuyoshi Shirai, Kazuaki Igarashi, Tadahiro Ozawa, Hiroshi Hagihara, Tohru Kobayashi, Katsuya Ozaki, and Susumu Ito. Ancestral sequence evolutionary trace and crystal structure analyses of alkaline α-amylase from bacillus sp. ksm-1378 to clarify the alkaline adaptation process of proteins. *Proteins: Structure, Function, and Bioinformatics*, 66(3):600–610, 2007. doi: 10.1002/prot.21255.

[44] Tsuyoshi Shirai, Vo Si Hung, Katsuhito Morinaka, Tohru Kobayashi, and Susumu Ito. Crystal structure of gh13 α-glucosidase gsj from one of the deepest sea bacteria. *Proteins: Structure, Function, and Bioinformatics*, 73(1):126–133, 2008. doi: 10.1002/prot.22044.

[45] Daniele Silvestro, Anna Kostikova, Glenn Litsios, Peter B. Pearman, and Nicolas Salamin. Measurement errors should always be incorporated in phylogenetic comparative analysis. *Methods in Ecology and Evolution*, 6(3):340–346, 2015. doi: 10.1111/2041-210X.12337.

[46] Lars K. Skov, Osman Mirza, Anette Henriksen, Gabrielle Potocki De Montalk, Magali Remaud-Simeon, Patricia Sarçabal, Rene-Marc Willemot, Pierre Monsan, and Michael Gajhede. Amylosucrase, a glucan-synthesizing enzyme from

the α-amylase family. *Journal of Biological Chemistry*, 276(27):25273–25278, 2001.

[47] Scott J. Steppan. Phylogenetic analysis of phenotypic covariance structure. i. contrasting results from matrix correlation and common principal component analysis. *Evolution*, 51(2):571–586, 1997. ISSN 00143820. URL http://www.jstor.org/stable/2411129.

[48] Stefan Strobl, Klaus Maskos, Georg Wiegand, Robert Huber, F. Xavier Gomis-Rüth, and Rudi Glockshuber. A novel strategy for inhibition of α-amylases: yellow meal worm α-amylase in complex with the ragi bifunctional inhibitor at 2.5 ă resolution. *Structure*, 6(7):911–921, 1998.

[49] Johan P Turkenburg, A Marek Brzozowski, Allan Svendsen, Torben V Borchert, Gideon J Davies, and Keith S Wilson. Structure of a pullulanase from bacillus acidopullulyticus. *Proteins*, 76(2):516–9, 2009. doi: 10.1002/prot.22416.

[50] Malene Bech Vester-Christensen, Maher Abou Hachem, Birte Svensson, and Anette Henriksen. Crystal structure of an essential enzyme in seed starch degradation: barley limit dextrinase in complex with cyclodextrins. *J. Mol. Biol.*, 403 (5):739–50, 2010. doi: 10.1016/j.jmb.2010.09.031.

[51] Keizo Yamamoto, Hideo Miyake, Masami Kusunoki, and Shigeyoshi Osaki. Steric hindrance by 2 amino acid residues determines the substrate specificity of isomaltase from *Saccharomyces cerevisiae*. *Journal of bioscience and bioengineering*, 112(6):545–550, 2011. doi: 10.1016/j.jbiosc.2011.08.016.

[52] Daohai Zhang, Nan Li, Shee-Mei Lok, Lian-Hui Zhang, and Kunchithapadam Swaminathan. Isomaltulose synthase (pali) of klebsiella sp. lx3 crystal structure and implication of mechanism. *Journal of Biological Chemistry*, 278(37):35428–35434, 2003.
