# Supplementary figures and images for: The response to selection in Glycoside Hydrolase Family 13 structures: A comparative quantitative genetics approach
Source: PLoS One. 2018 Apr 26;13(4):e0196135. doi: 10.1371/journal.pone.0196135 (PMC5919626; doi:10.1371/journal.pone.0196135)

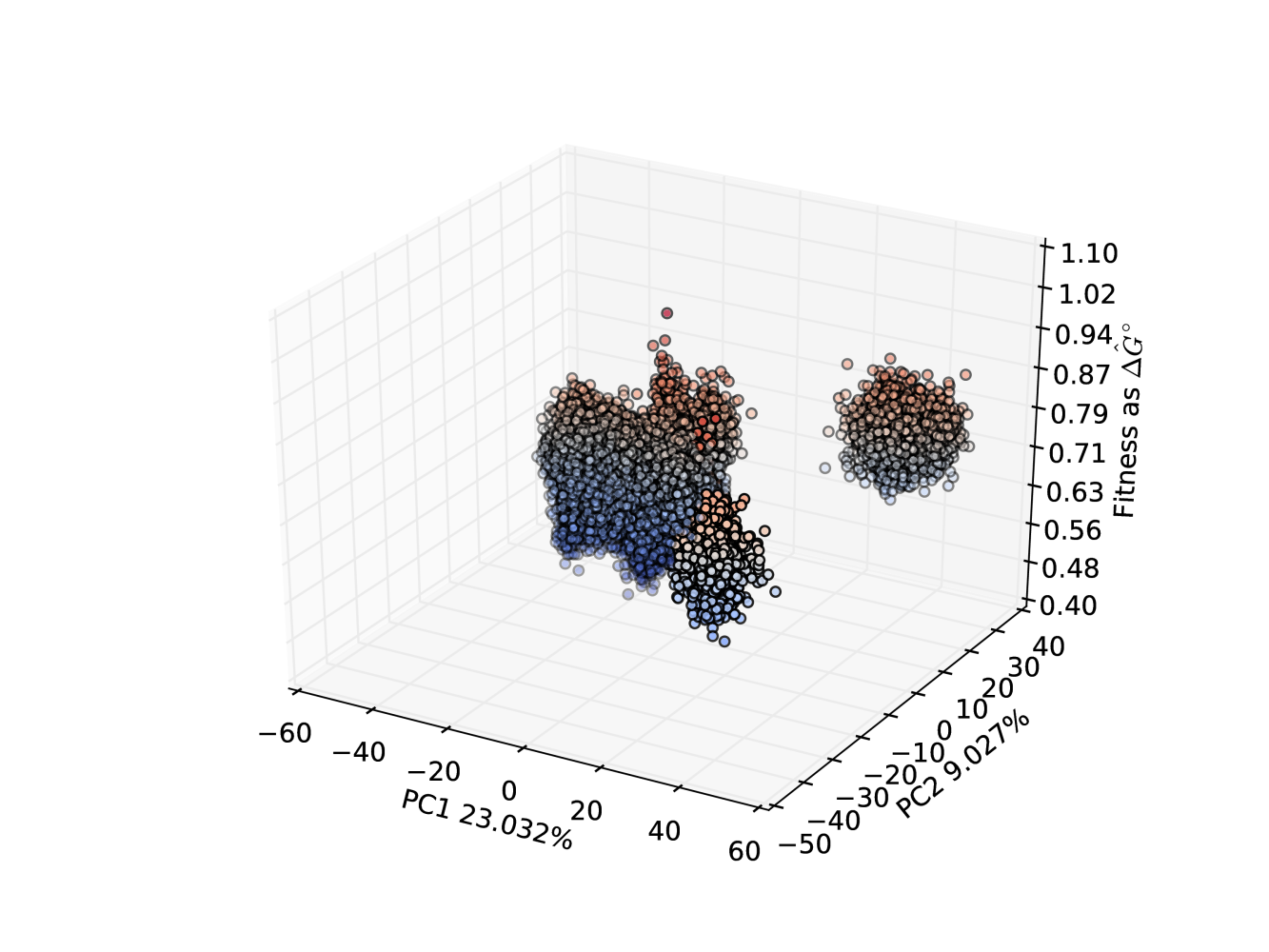

Supplement: S1 Fig — Fitness in the Z axis is defined as ΔG°^. (PNG) [file pone.0196135.s002.png]
